# Ultrasound neuromodulation depends on pulse repetition frequency and can modulate inhibitory effects of TTX

**DOI:** 10.1038/s41598-020-72189-y

**Published:** 2020-09-18

**Authors:** Thomas J. Manuel, Jiro Kusunose, Xiaoyan Zhan, Xiaohui Lv, Ellison Kang, Aaron Yang, Zixiu Xiang, Charles F. Caskey

**Affiliations:** 1 Vanderbilt University Institute of Imaging Science, Nashville, TN USA; 2 Department of Radiology and Radiological Sciences, Vanderbilt University Medical Center, Nashville, TN USA; 3 Department of Biomedical Engineering, Vanderbilt University, Nashville, TN USA; 4 Department of Pharmacology, Vanderbilt University, Nashville, TN USA; 5 Vanderbilt Center for Neuroscience Drug Discovery, Vanderbilt University, Nashville, TN USA

**Keywords:** Biomedical engineering, Preclinical research

## Abstract

Ultrasound is gaining traction as a neuromodulation method due to its ability to remotely and non-invasively modulate neuronal activity with millimeter precision. However, there is little consensus about optimal ultrasound parameters required to elicit neuromodulation and how specific parameters drive mechanisms that underlie ultrasound neuromodulation. We address these questions in this work by performing a study to determine effective ultrasound parameters in a transgenic mouse brain slice model that enables calcium imaging as a quantitative readout of neuronal activity for ultrasound neuromodulation. We report that (1) calcium signaling increases with the application of ultrasound; (2) the neuronal response rate to ultrasound is dependent on pulse repetition frequency (PRF); and (3) ultrasound can reversibly alter the inhibitory effects of tetrodotoxin (TTX) in pharmacological studies. This study offers mechanistic insight into the PRF dependence of ultrasound neuromodulation and the nature of ultrasound/ion channel interaction.

## Introduction

Ultrasound neuromodulation (USN) is gaining traction as a non-invasive neuromodulation modality but little is known about how ultrasound affects neurons. Ultrasound (US) can alter the behavior of the nervous system, and researchers have demonstrated these effects sporadically throughout the past century in different in vivo and ex vivo preparations^1,2^. Since this time, researchers have routinely validated USN in animal models such as in vivo murine^3,4^, rabbit^5^, sheep^6^, and non-human primate^7^. Researchers have recently found ultrasound stimulation of specific regions of the primate brain elicits responses in both the stimulated and connected regions^8,9^. Ultrasound offers a potential therapy in these cases by enabling non-invasive modulation of specific brain circuit nodes that underlie diseases such as disorders of consciousness^10^, chronic pain^11^ and Alzheimer’s disease^12^. For an in-depth review on USN results, mechanisms, and safety see Blackmore et al.^13^. Further information about the interactions of ultrasound with neurons will be crucial to fully leverage this therapeutic technology.

As ultrasound propagates through tissue it displaces particles and can potentially generate biological effects through mechanical or thermal effects^14^. At low mechanical indexes where diagnostic imaging occurs, the tissue is mostly unaffected and returns to its original state after the ultrasonic wave propagates. As the mechanical index is increased, the displacement can be large enough to generate direct mechanical effects or heat. Although heat is known to affect neural activity at the bulk and molecular scale^15,16^, USN is frequently observed using power levels that do not generate significant heat (< 0.1 °C)^17^. We only explore pulses in this “non-thermal” regime in the present study.

There is increasing evidence that mechanical effects of ultrasound underlie neuromodulation, yet the discussion remains open. Ye et al. found that mechanical index correlates with response frequency in mice, directly linking particle displacement to USN^18^. A study in *C. elegans* showed that mutants without thermal sensitivity responded to US while mutants without mechanical sensitivity did not^19^. This mechanical effect may be manifesting via ion channels. Researchers observed ion currents in bi-layer preparations containing the Nav1.2 ion channel during sonication^20^. Nikolaev et al. found that pyramidal neurons express stress activated cation channels that trigger action potentials when subjected to pressure^21^. In the Xenopous oocyte, ion channel current modulation dependence on US power has been reported^22^. Prieto et al. showed activation of the Piezo1 channel by 43 MHz ultrasound and reported acoustic streaming, or the displacement of fluid in the direction of ultrasound propagation, rather than particle displacement as the primary mechanism^23^. An alternative transduction mechanism excluding ion channels is that the pressure-induced displacement of the lipid bi-layer generates action potentials^24^. By extending the Hodgkin and Huxley model to include capacitance changes in the cell membrane due to ultrasound, Plaksin et al. created a model^25^ that matches well with in vivo observations of mouse motor cortex activation via ultrasound^26^.

In implementing USN, several parameters can be varied for pulse design. These include fundamental frequency, duty cycle, pressure, and pulse repetition frequency (PRF). Since mechanisms remain unknown, researchers select parameters using empirical and ad hoc methods. An early study detailing the use of ultrasound for neuromodulation employed PRF in the kilohertz range^27^. Many studies have since chosen a similar repetition frequency in USN pulses presumably based on this work, but there is little physiological basis for introducing this pulsed scheme^26,28,29^. King et al. did not find PRF to increase stimulation success, while others have noted that burst parameters elicit a strong off-target auditory effect which can confound the direct neuromodulatory effect^30,31^. Yoon et al. conducted a thorough parametric study in sheep and found continuous ultrasound to perform worse than pulsed ultrasound for cortical and thalamic stimulation^32^. Other studies investigating USN in peripheral nerves show that tuning PRF affects skin sensations and readouts from functional magnetic resonance imaging (fMRI) and electroencephalographic (EEG) data^33,34^. As our understanding of direct neuromodulatory and off-target effects evolves, increased knowledge about the nature of mechanical stimulation is desirable to clarify the role of PRF and help us design pulses that are optimized for neuromodulation.

In order to improve our understanding of the ultrasound parameters that best modulate neurons, we quantified neuronal activity in an ex vivo brain slice model using a range of non-thermal ultrasound parameters. This manuscript describes the methods and experiments used to test USN parameters in a murine brain slice model using calcium imaging for activity measurement. These measurements are independent of artifacts from auditory pathway confounds as well as artifacts from ultrasound interacting with electrodes. We show direct observation of calcium signaling in response to ultrasound at parameters reported by others. Furthermore, we show dependence of USN on PRF, revealing that tuning PRF affects response rates. Using low concentration inhibition agents, we demonstrate that pulsed ultrasound reversibly affects ligand/channel kinetics, highlighting a potential mechanism that has not been previously considered. Our observations provide an important link between observations at the single cell and whole animal.

## Results

### Calcium signaling increases following ultrasound

**Figure 1:**
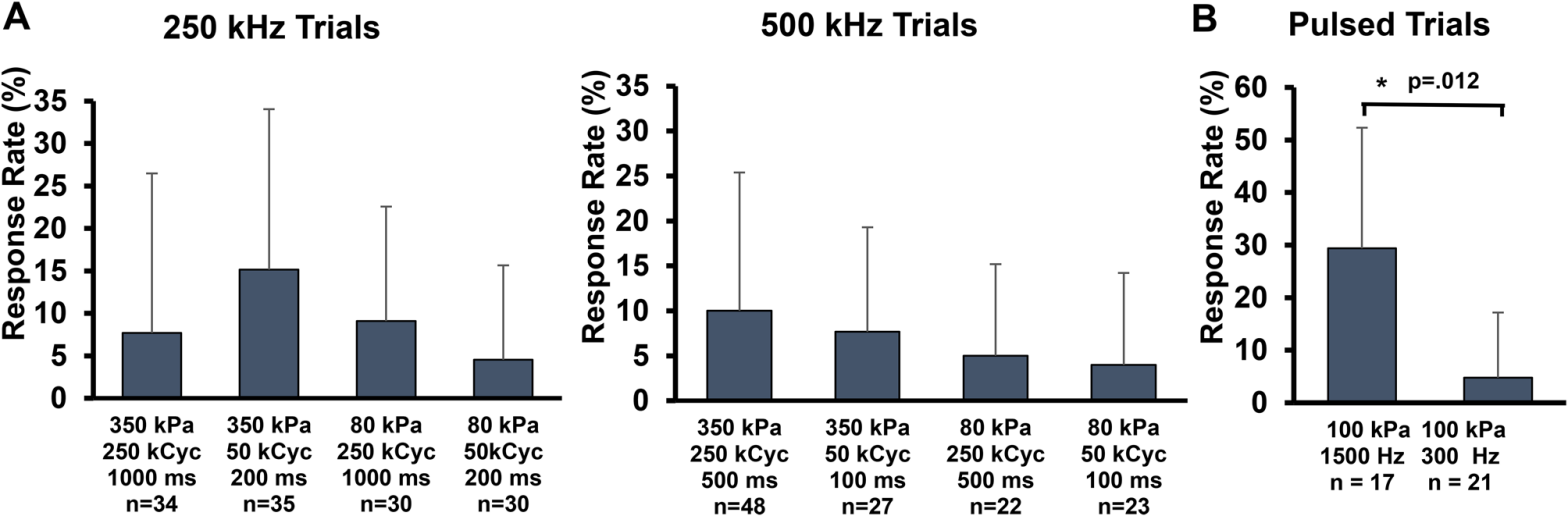
Response rates for all investigated parameters reporting average and standard deviation across slices. (**A**) 250 and 500 kHz continuous wave trials varying pressure and pulse duration. Pulse duration is half for 500 kHz trials because the number of pressure cycles were matched across frequency. (**B**) 500 kHz pulsed ultrasound trials at 1,500 Hz and 300 Hz PRFs with duty cycle, intensity, and frequency matched. See Fig. 2 for pulsed parameter details. *kPa* kilopascals (pressure), *kCyc* kilocycles (number of pressure cycles). *Student’s paired t-test.

Brain slices were sonicated with 8 continuous wave parameters with varying sonication frequency, pressure, and pulse length. Sample size (n) refers to number of trials where a trial comprises a baseline measurement, sonication measurement, and a rest period of 30 s or greater. Two pulse lengths were tested for 250 kHz and 500 kHz with a matched number of cycles. The shorter pulse was 50 kilocycles of sound, which corresponds to 200 ms and 100 ms for 250 kHz and 500 kHz, respectively. The longer pulses were 250 kilocycles, which were 1,000 ms and 500 ms for 250 kHz and 500 kHz sound, respectively. We matched the number of cycles to account for frequency dependent differences in thermal deposition between 250 and 500 kHz. Continuous wave ultrasound increased calcium signaling in brain slices in 19 out of 221 total trials (8.5%) across 53 slices (Fig. 1A). For the continuous wave parameters examined (80 and 350 kPa, and 50 and 250 kilocycles), the response rate was less than or equal to 15%. Among these parameters, 200 ms 350 kPa 250 kHz pulses showed the highest average response rate across all slices (5 out of 33 trials, 15%) but this was not statistically significant compared to the other parameters. Brain slices were sonicated with two pulsed ultrasound parameters with duty cycle, intensity, and transmit frequency held constant and PRF of 1,500 Hz and 300 Hz (Fig. 1B). When analyzing these trials using the same criteria for continuous pulses over a time frame encompassing a matched number of ultrasound cycles, we found that the response rate was 29% and 5% for 1,500 and 300 Hz PRFs, respectively (p = 0.012, Student’s t-test).

### Neuronal response is PRF dependent

**Figure 2:**
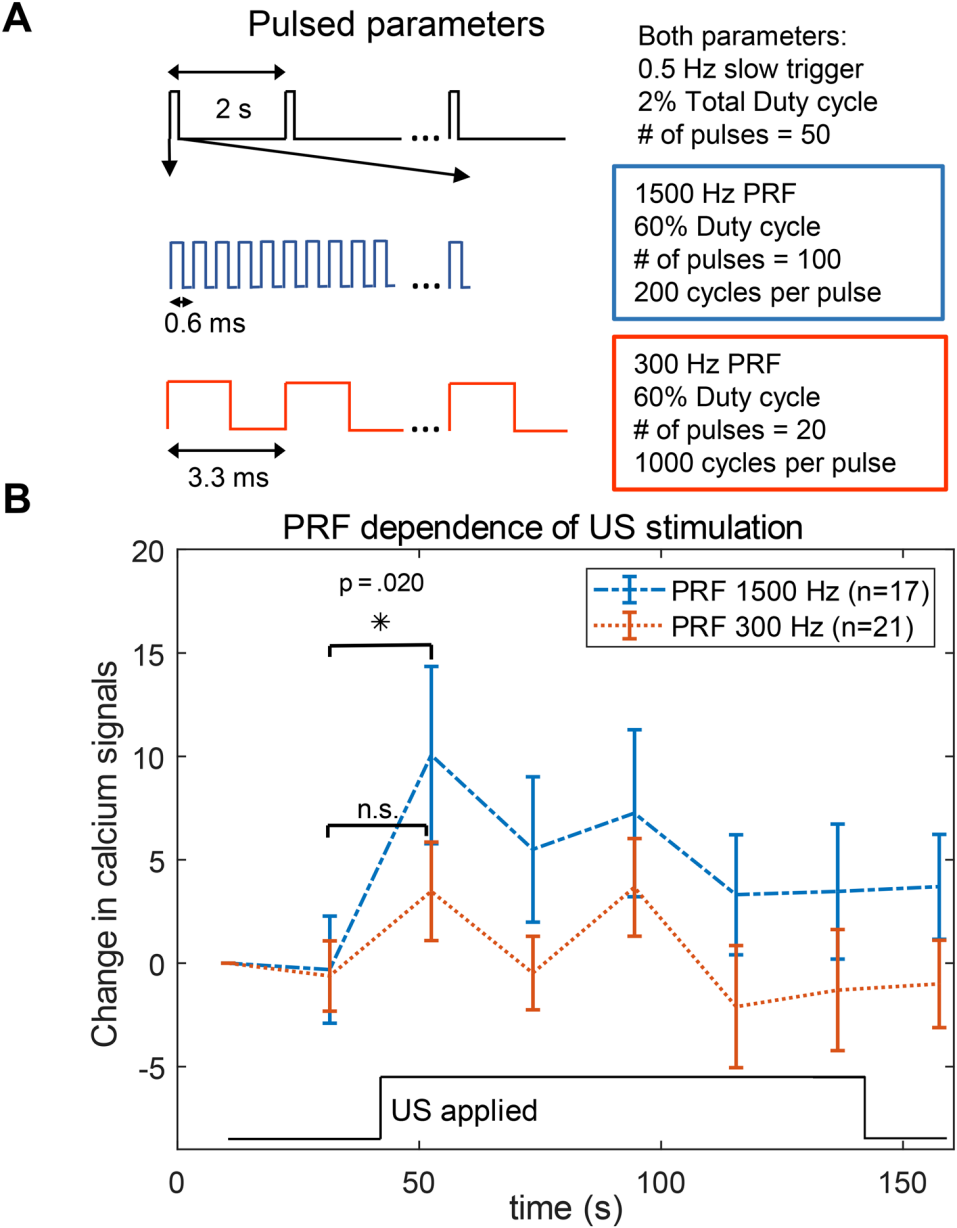
Pulse repetition frequency affects calcium signaling rates. (**A**) US parameter details. A slow trigger at 0.5 Hz which fired 50 times was used for both parameters. This trigger activated the two PRFs shown in blue and red which varied in pulse length and number of pulses to enable matched duty cycle. (**B**) Calcium signaling at two PRFs with duty cycle and power matched. Only PRF 1,500 Hz in the time bin immediately following US onset shows significant increase from baseline signaling (*p = 0.02, student’s paired t-test). Data are presented as mean ± SEM. (*n.s.* not significant).

To investigate the effect of PRF on neuronal responses, slices were sonicated at two PRFs with duty cycle, intensity, and transmit frequency matched (Fig. 2). We chose 1,500 Hz PRF with 500 kHz frequency because similar parameters have elicited measurable responses in humans and non-human primates along with minimal induced heating^8,29,35^. 300 Hz PRF has been previously reported to be less effective than 1,500 Hz in mice^26^ but equally effective in *C. elegans*^19^. The duty cycle was 60% during bursts and 2% for the total sonication which includes inter-burst intervals. The total sonication time was 100 s (50 bursts, 0.5 Hz). 1,500 Hz PRF resulted in an increase in calcium signaling from the baseline (p = 0.02, student’s t-test) for the time point immediately following US onset. No timepoints from 300 Hz trials showed statistically significant change from baseline. At each timepoint during sonication, 1,500 Hz trials induced a greater change in calcium signals than 300 Hz trials. These differences were not statistically significant (p ≥ 0.13). Duty cycle, intensity, and transmit frequency were held constant because they have each been shown to affect US neuromodulation^18,26,32^.

### Ultrasound modulates ion channel interactions

**Figure 3:**
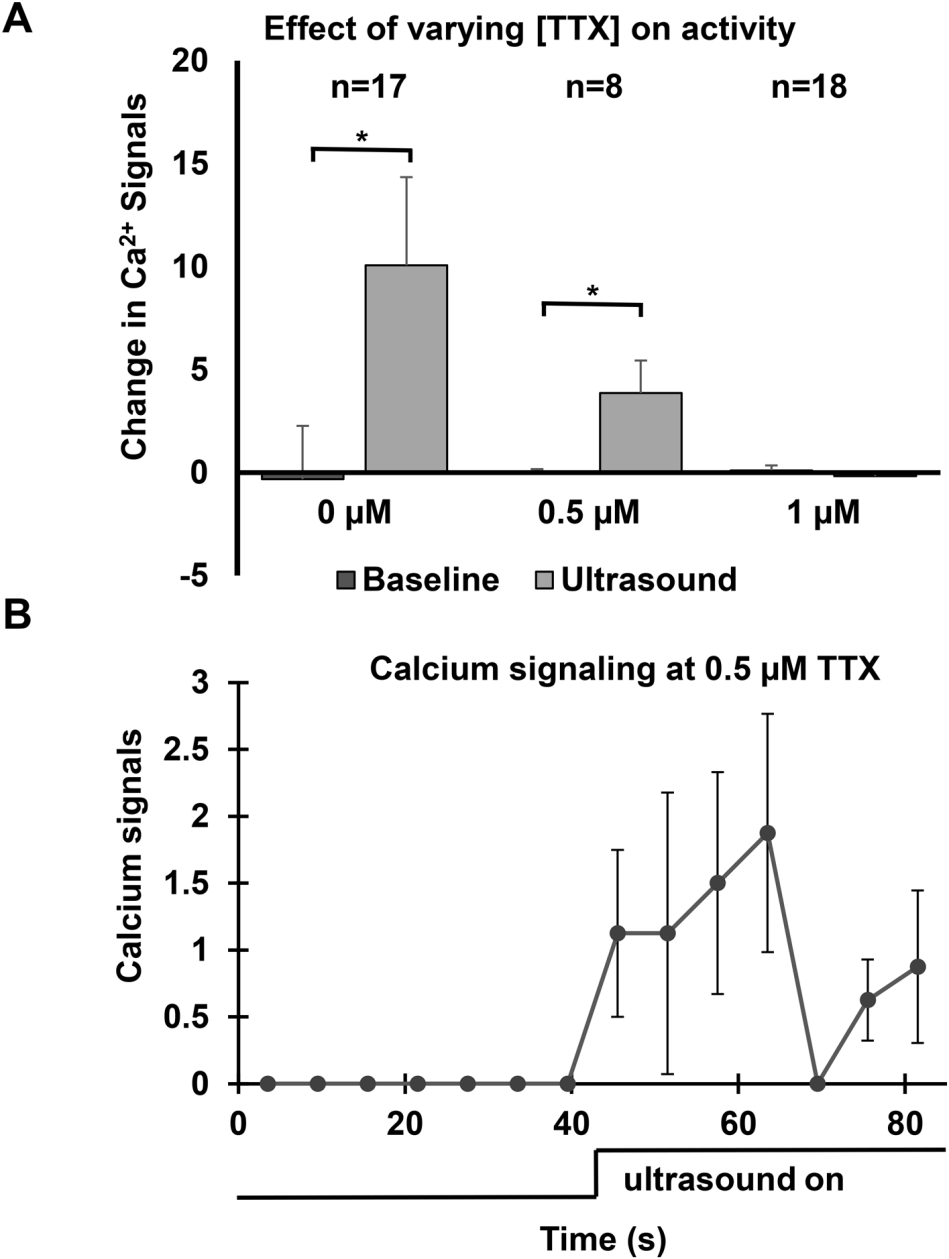
(**A**) Change in calcium signaling during ultrasound with three concentrations of the voltage-gated sodium channel blocker, TTX. At 0.5 µM TTX, ultrasound temporarily reduces the inhibitory effect of TTX. (*p < 0.05, student’s paired t-test). (**B**) Calcium signals vs. time at 0.5 µM TTX showing reduction of TTX inhibition during ultrasound (n = 8). All data are presented as mean ± SEM.

Addition of 1 µM TTX eliminated baseline spontaneous as well as US-induced calcium mobilization (Fig. 3). At a reduced concentration of 0.5 µM TTX, baseline calcium signals were eliminated, but US induced calcium signaling. During 0.5 µM TTX tests, we measured two brain slices with 4 observations in each slice and 2 min of rest between trials. Increased Ca^2+^ signaling in the presence of 0.5 µM TTX only occurred during sonication and returned to baseline after sonication. As a positive control, we compared spontaneous baseline activity between no TTX and 0.5 µM TTX, with the expected outcome being suppression of calcium signaling at 0.5 µM TTX. In the absence of TTX, calcium signaling during baseline was 1.1 signals per second compared to 0.0 signals per second for 0.5 µM TTX (p = 0.0007, student’s t-test). Pulsed ultrasound was associated with increased Ca^2+^ signaling at the 0.5 µM concentration of TTX that fully blocked Ca^2+^ signaling at baseline.

### USN pulses generated limited heat and displacement

Beam maps reporting relative acoustic intensity measured in a waterbath at the face of both reflector cones had uniform pressure within the microscope field of view. For continous wave pulses used in Fig. 1, heating from acoustic absorption was less than 1 °C at the maximum pressure and pulse duration used. For pulsed ultrasound, heating was less than 0.25 °C. There was no detectable change in image intensity due to displacement from the acoustic radiation force imparted on the brain slices for parameters reported in this study.

## Discussion

USN has been demonstrated in multiple experimental models, but there are many confounds that can make interpreting experimental outcomes challenging. Our study demonstrates direct USN in a brain slice model using optical imaging for feedback. By using genetically targeted optical methods to image neural activity, our reported measurements are isolated from off-target effects or other known artifacts. We report overall success rates using non-thermal parameters known to elicit neuromodulation in various animal models and demonstrate that USN is PRF dependent and capable of modulating ion channel interactions with pharmacological agents. Our observations provide an important link between single cell experiments and work in fully intact brains.

### Optical readouts avoid potential confounds

Using optical methods to assess neural responses to ultrasound avoids potential confounds created by the presence of an electrode. Traditional electrophysiology is challenging in the presence of ultrasound because electrodes are typically small metal probes which are highly absorbing and scattering as reported by Morris et al.^36^. When an ultrasound pulse propagates, it generates a force that is proportional to the absorption (α) of the propagating medium and intensity (I) of the pulse given by:

$$F=\frac{2\alpha \mathrm{I}}{c}$$

with c as the speed of sound in the media^37^. At non-thermal ultrasound pressure used for neuromodulation, this force is on the order of µN/cm^3^ to mN/cm^3^ in brain tissue, but the addition of a highly absorbing and scattering electrode causes a stronger force to be imparted. The induced motion of the electrode would result in both viscous and absorptive heating^36^ amplifying the mechanical effects of ultrasound and confounding any measurements. Electrodes can also result in standing pressure waves which alter the distribution of pressure and radiation force in surrounding tissue^38^. Ultrasound-induced artifacts have also been reported in patch clamping methods using glass pipette electrodes due to a disruption of the connection between the tissue and probe^39^. Optical imaging, as used in our study, mitigates these confounds present in electrophysiology, although we note that radiation force interactions in the slice preparation differ from the intact brain in two main ways. When used at sufficient pressure, the acoustic radiation force can displace the tissue slice out of the focal plane, generating false positive readings. The pressures used in our study did not displace the tissue by a detectable amount. Acoustic streaming is the displacement of fluid in the direction of ultrasound propagation. In our slice preparation, which is acoustically similar to Prieto et al., acoustic streaming directly at the Thermanox layer would be zero and increase with increasing distance away from the Thermanox layer^23^. Streaming at the slice location (directly above the Thermanox) was not strong enough to generate detectable displacement at pressures used in our study. However, the overall fluid dynamics of acoustic streaming likely differ between the brain slice and intact brain. Models of acoustic propagation in the brain that incorporate this effect do not exist and would improve our understanding of streaming in the intact brain.

Optical tracers for readouts within the ultrasound pressure field remain the most non-invasive and essential tool for USN measurements. For this reason, Lee et al. have developed a system for optical readouts while sonicating co-cultured neurons and astrocytes^40^. Other groups have used optical tracers in vivo including Han et al. who showed that ketamine blocks USN in in vivo cortical neuron activity using calcium imaging with indicator OGB-1 AM^41^ and Sato et al. who used wide-field cortical imaging with GCaMP6s to monitor US neuromodulation in vivo but report no observation of direct US neuromodulation^31^. The isolated brain slice in our study targeted GCaMP6s in a similar manner but shows direct neuromodulation from ultrasound.

### Direct neuromodulation in the absence of auditory confounds

The ability for ultrasound to elicit audible sensations in humans was reported in studies as early as 1950 (Pumphrey, 1950). The precise mechanism through which ultrasound activates the auditory system is not fully understood and is hypothesized to involve mode conversion of the ultrasonic wave into shear waves within the bone (Clement et al. 2004) or coupling through the cochlear fluid^30^. A prior study in the intact mouse reported no evidence of direct stimulation with widefield calcium imaging during transcranial stimulation of mice expressing GCaMP6s proteins in neurons bearing the Synapsin I promoter^31^. We used similar genetic targeting to this prior work but observed direct effects that were not observed in the intact animal. We hypothesize that Sato et al. observed a combined effect of direct USN and auditory effects but the direct effects were below the detection threshold of the in vivo optical system, which would primarily be sensitive to cortical activation in the living animal. This interpretation is consistent with in vivo studies in genetically deafened mice that demonstrate motor responses from transcranial ultrasound^42^.

### Role of PRF

Continuous and pulsed wave ultrasound have been shown to elicit a wide range of neuromodulatory effects in a variety of animal models (for reviews see Tufail et al.^27^ and Blackmore et al.^13^). The use of pulsed bursts in the kilohertz range generates acoustic waves capable of generating auditory brainstem response in mice, which can be mitigated by using smooth amplitude windows for the modulatory wave to reduce audible frequency components^42^. The inclusion of a pulsed wave compared to continuous ultrasound was not a strong indicator for modulation success in a mouse study measuring motor responses to modulation of the motor cortex^26^. However in *C. elegans*, Kubanek et al.^19^ reported maximum frequency of motor responses at PRFs between 300 Hz and 3 kHz and 50% duty cycle using 10 MHz ultrasound. In a study sonicating the motor cortex of rats, Kim et al.^28^ report that pulsed ultrasound elicits responses at lower acoustic intensities thresholds than continuous wave ultrasound (PRFs up to 2 kHz with varying duty cycle were investigated). Our results agree with these findings, as we report that 1,500 Hz PRF low pressure, pulses with 60% intra-burst duty cycle and 2% total duty cycle is effective.

It is interesting to consider the temporal aspects of tissue displacement during pulsed ultrasound. With the 1,500 Hz PRF, 200 cycle, 500 kHz, pulses used in this study, the relaxation time between single pulses is 260 µs. Using Viscoelastic Response (VisR) imaging^43^, an acoustic radiation force based elastography method which employs multiple displacement pulses to infer mechanical properties of tissue, a relaxation time of 240 µs is employed between pulses to allow for partial tissue relaxation. Their model shows that tissue relaxation occurs at timescales similar to the off periods of PRFs which have incidentally become popular in USN. With tissue relaxation occurring in the off time of these pulses, it follows that tuning PRF and duty cycle is equivalent to tuning the displacement and relaxation dynamics of sonicated tissue. If ARF induced displacement is the predominant transduction mechanism for USN—as suggested in^38^—it follows that tuning the temporal displacement profile could result in varied response rates due to ultrasound. In our study, pulsed ultrasound at a PRF of 1,500 Hz exhibited robust response. The mean change in calcium signals was higher in every sonication time bin compared to 300 Hz PRF, with duty cycle and pressure held constant. The use of pulsed ultrasound enables neuromodulation at low duty cycles, making it a desirable candidate for in vivo applications where heating from absorption should be minimized.

### Continuous wave trials showed low response rates

The response rates across continuous wave trials were low (< 15%) and less robust than pulsed ultrasound in this model. When analyzing pulsed ultrasound trials with the same protocol used for continuous wave pulses, the 1,500 Hz PRF pulse resulted in 29% response rate at lower pressure (100 kPa in pulsed trials versus 350 kPa in continuous wave trials). This metric only included the 24 s following the onset of ultrasound in pulsed trials to keep the total amount of pressure cycles delivered comparable (250 kilocycles in continuous wave trials, 240 kilocycles in pulsed trials). The response rate is much lower than what is reported in in vivo murine models where motor responses > 80% were found for very similar US parameters (500 kHz 80 ms pulses at 300 kPa or 2.9 W/cm^2^ I_SPTA_)^42^. Factors inherent to our experimental design may have contributed to these low response rates. The magnitude of the calcium response must be high enough to distinguish spontaneous activity from US induced activity. Tissue scattering limits the depth of the 300 µm thick brain slice which is resolvable by the microscope, meaning only neurons in the top portion of the slice contribute to measured signals. The number of neurons exposed to the US is inherently lower in the slice than in vivo given that the slice occupies only a small portion of the US focus as opposed to in vivo*,* where the entire sound focus may interact with a large population of neurons in brain tissue. A similar explanation relating exposure volume to stimulation is offered in both Ye et al.^18^ and Menz et al*.*^38^ Furthermore, several in vivo studies explore higher pressure regimes for USN. In our model pulses above 350 kPa often resulted in slice motion which limited our ability to explore higher pressure.

### Inhibitory effects of low concentration TTX on calcium mobilization are temporarily reduced by pulsed US

Our model demonstrated a complete blocking of baseline calcium signaling at 1 uM TTX and a lack of response to ultrasound at that concentration. This agrees with the findings of Tyler et al.^39^ who showed at 0 to 100 Hz PRF, 440 kHz US that 1 µM TTX suppressed US stimulation and Lin et al.^44^, who demonstrated inhibition in the presence of continuous wave 27 MHz US with 0.1 µM TTX in pyramidal cells of rat brain slices using whole-cell patch-clamp recordings. These findings suggest that US stimulates neurons through a transduction pathway influenced by voltage-gated Na^2+^ channels. Voltage-gated Na^2+^ channel conductance has been shown to increase with mechanical deformation^45^, providing a potential mechanism for US ion channel interaction. Gaub et al. found that mechanical deformation of neurons with pressures greater than 6 kPa resulted in increased calcium signaling in cultured cortical and hippocampal mouse cells expressing GCaMP6s and suggest sub-traumatic pressures applied to neurons evoke neuronal responses via gating of ion channels^46^. Unique to our results, a concentration of 0.5 µM TTX suppressed the baseline level of calcium signaling, but US still induced calcium mobilization. In these trials, ultrasound temporarily reduced the inhibitory effect of TTX. We hypothesize that ultrasound reversibly alters the interaction of TTX at 0.5 µM with voltage-gated Na^2+^ channels of pyramidal cells.

## Conclusion

Pulsed ultrasound at a PRF of 1,500 Hz increased calcium signaling in neurons, confirming the efficacy of this parameter and that low duty cycle low intensity ultrasound can be used to directly excite neurons. Pulsed ultrasound is more effective for USN than continuous wave ultrasound in this model. This finding is encouraging for transcranial applications where pulsed ultrasound is conducive for higher pressure and lower tissue heating. Our findings offer further insight into sodium channel involvement in US neuromodulation by demonstrating that US can reduce the inhibitory effect of TTX on voltage gated sodium channels.

## Methods

We used a calcium imaging brain slice model to optically measure intracellular calcium mobilization of genetically tagged neurons^47^ in response to ultrasound. Coronal brain slices containing the motor cortex were prepared from transgenic mice selectively expressing genetically encoded calcium indicator GCaMP6s in cortical pyramidal cells or all neuronal cells by crossing Cre-dependent GCaMP6s mice (JAX #024106) with CaMKIIα-Cre mice (JAX #005359) or Syn-Cre mice (JAX #003966), respectively. Animals were housed under a 12-h light/dark cycle with free access to food and water in their home cages. All procedures were approved by the Institutional Animal Care and Use Committee of Vanderbilt University and conformed to the guidelines established by the National Research Council, the *Guide for the Care and Use of Laboratory Animals.* In brief, mice (both male and female, 6–19 weeks of age) were anesthetized with isoflurane, euthanized, and decapitated. Brains were rapidly removed and submerged into oxygenated (95% O_2_ /5% CO_2_), ice-cold NMDG-based cutting/recovery solution (in mM: 93 NMDG, 2.5 KCl, 1.2 NaH_2_PO_4_, 30 NaHCO_3_, 20 HEPES, 25 d-glucose, 5 sodium ascorbate, 2 thiourea, 3 sodium pyruvate, 10 MgSO_4_, 0.5 CaCl_2_; pH 7.3, 298–305 mOsm). Coronal slices (200–300 μm thick) containing the motor cortex were cut using a Leica VT1200S microtome (Leica Microsystems Inc, Wetzlar, Germany) and transferred into and incubated in a chamber containing the NMDG-based cutting/recovery solution aerated with 95% O_2_/5% CO_2_ at 32 °C for 8 min. Slices were then maintained at room temperature in a holding chamber containing artificial cerebral spinal fluid (aCSF) (in mM: 126 NaCl, 2.5 KCl, 1.25 NaH_2_PO_4_, 2 CaCl_2_, 1 MgSO_4,_ , 26 NaHCO_3_ and 10 d-glucose) for at least 1 h until transferred to an imaging chamber superfused with oxygenated aCSF.

### Setup

**Figure 4:**
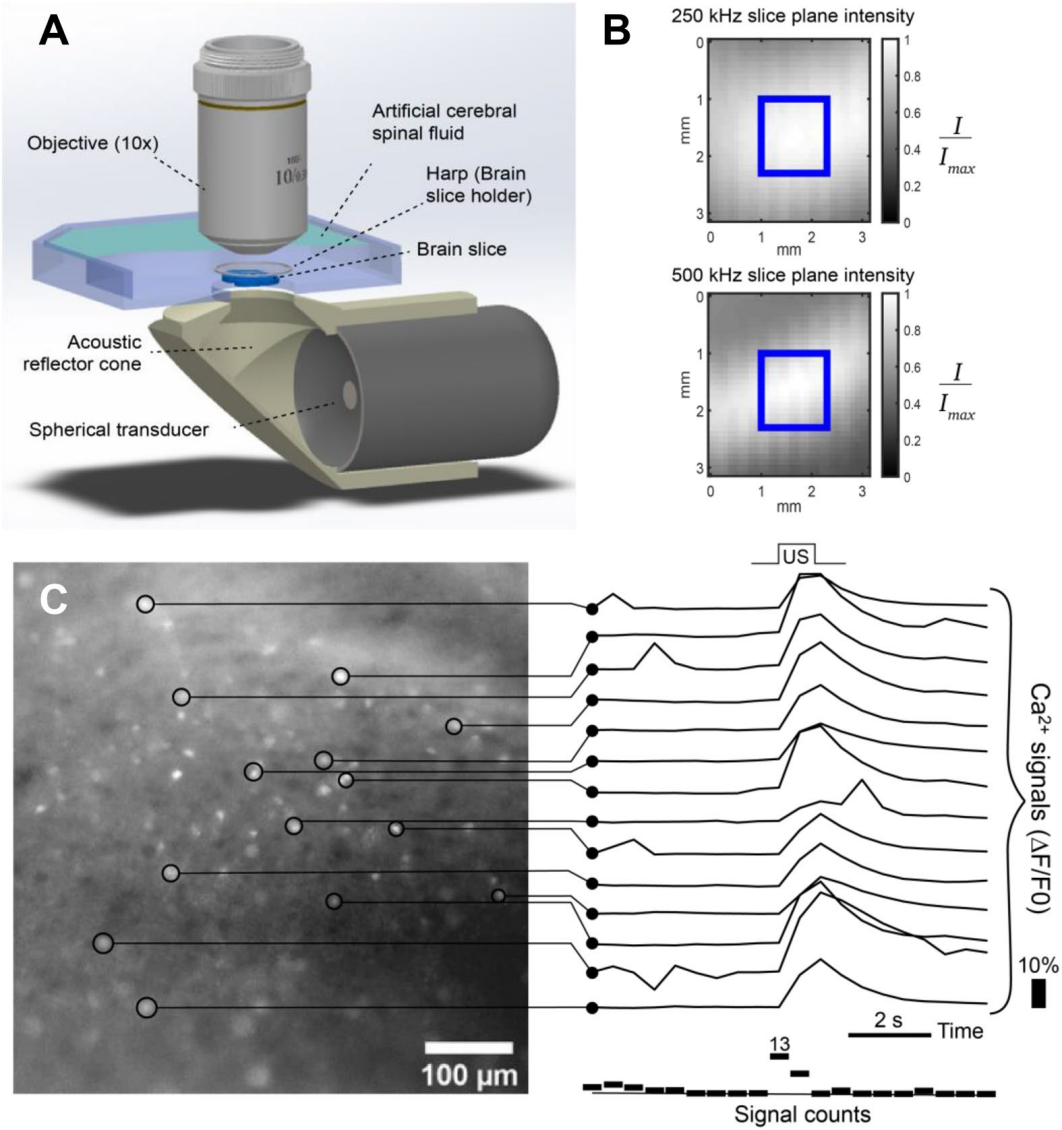
(**A**) Experimental setup showing sonication from below while imaging from above. (**B**) Relative acoustic intensity at the plane of the slice (blue square marks field of view of microscope, 1.3mm^2^). (**C**) Processing procedure showing Ca^2+^ signal traces for individual ROIs. Ca^2+^ images (left) taken at 2 frames per second. Number of signal increases for each frame is shown below the traces. Response to US was determined by comparing signal rates between baseline frames and frames during and after US stimulation.

We developed an experimental apparatus capable of delivering ultrasound to a brain slice via a 1-inch spherically focused transducer (NDT, Huntington Beach, CA, USA) of either 250 kHz or 500 kHz (Fig. 4A) center frequency powered by an amplifier and function generator (A150, E&I, Rochester, NY, USA; Keysight, 33500B Santa Rosa, CA, USA)**.** Figure 4A was rendered using Solidworks (Solidworks Corp., Waltham MA). Sound was delivered through an agarose filled custom 3D printed acoustic reflection cone coupled through a Thermanox membrane (Nalge Nunc, Rochester, NY, USA) and into an imaging chamber superfused with oxygenated aCSF (34 °C). The cone was designed so that the propagation direction was not perpendicular to the microscope objective to reduce standing wave effects. The brain slices were held in place above the acoustically transparent membrane in the imaging chamber by a harp (Warner). The harp strings which are 40 µm in diameter were positioned such that they were not within the field of view of the microscope and were thus not affecting the ultrasound path through the neurons being imaged. Fluorescent images were captured using an Olympus BX50WI upright fluorescence microscope equipped with a 10 × water immersion objective (Olympus, Lake Success, NY). Blue light (470 nm LED, Thorlabs Inc., New Jersey) was delivered through the 10 × water immersion objective lens on the microscope. Clampex software (Molecular Devices, San Jose, CA) and HCImage Live (Hamamatsu, Japan) were used for triggering and image acquisition. A Hamamatsu ORCA-Flash4.0 LT digital camera (Hamamatsu, Japan) was used, sampling at 6.5 µm per pixel and 2 frames per second with a 1.3 mm field of view.

On each day of experimentation, the acoustic reflector cones (one for each frequency tested) were filled with fresh agarose solution and allowed to set for 1 h. The pressure output was measured for both cones by coupling the cone face to a waterbath and measuring pressure with a ceramic hydrophone (Onda, Sunnyvale, CA). The pressure maximum for each cone was marked by crosshairs on the cone face, which were used as a reference to accurately position the motor cortex of the brain slices in the imaging chamber. The uniformity of the acoustic intensity across the microscope field of view is shown for both cones in Fig. 4B. Temperature measurements were recorded once for each US parameter reported using a thermocouple (MAX31855, Adafruit, New York, NY USA) placed at the hotspot in the imaging chamber and read by an Arduino UNO (Arduino, Somerville, MA, USA).

### Protocol for ultrasound calcium imaging trials

Slices were transferred to the imaging chamber and allowed to rest for 2 min prior to imaging. A ‘single trial’ is a measurement which includes calcium imaging during a baseline period and calcium imaging during a sonication period. For continuous wave experiments 30 s of images were acquired at 2 Hz with 20 s baseline and 10 s post sonication. For low duty cycle trials varying PRF, longer acquisitions were used with 40 s of baseline followed by 120 s post sonication. The minimum time between repeated trials was 30 s.

### Processing image sequences

The general processing approach is shown in Fig. 4C. Processing was done in MATLAB R2019a (MathWorks, Natick, MA) and ImageJ^48^. Each dataset was corrected for photobleaching by fitting an exponential model to the average intensity over time. Cell ROIs were selected manually using the ImageJ oval tool while visualizing the signal change (∆F/F_0_) which allowed recording individual cells that were active during the entire observation window. These cell ROIs were loaded into MATLAB to generate fluorescent plots. An individual Ca^2+^ signal was defined as a change in an ROI’s mean intensity by > 1% over the span of 2 or less frames (1 s).

### Continuous wave trials

The signaling rates for baseline frames and sonication frames were calculated by counting all signals within those frames and dividing by the number of frames. The signaling rate represents the calcium signals per time and allows quantification of calcium signaling increase during sonication. We ensured that slices were healthy by rejecting observations with low slice activity (< 0.2 signals per frame inclusive of baseline and post-sonication time frames). To account for the range of number of active cells and level of spontaneous activity exhibited in slices, a slice was considered as responsive to US if the signal rate increased by either 90% or 0.4 signals per frame compared to its baseline. Two metrics were used because a trial with few active cells and low baseline activity (~ 0.3 signals per frame) is unlikely to increase by 0.4 signals per frame, even given a response but is capable of a 90% rate increase. Conversely, a slice with many active cells and a high baseline activity (~ 1.5 signals per frame) may exhibit increases of 0.4 signals per frame given a response but will not increase by 90% as that would require a rate of 2.8 signals per frame.

### Pulsed ultrasound trials

The trials investigating PRF differed from continuous wave trials in that they were lower duty cycle (2% total duty cycle and 60% burst duty cycle as opposed to 100% in continuous wave) and longer in duration (160 s vs. 30 s). A center frequency of 500 kHz and pressure of 100 kPa were used. Both pulsed parameters used a slow trigger at 0.5 Hz which activated the bursts 50 times per trial. To match duty cycle while varying PRF, pulse length (number of cycles) and number of pulses per burst were varied. The 1,500 Hz pulses used 200 cycles per pulse (0.4 ms) and 100 pulses per burst. The 300 Hz pulses used 1,000 cycles per pulse (2 ms) and 20 pulses per burst. Firing rates were grouped into time bins with 20 s duration. To account for variability in spontaneous activity from trial to trial, signaling rates were offset by the first baseline bin so that each bin represents the change in calcium signaling rate during the trial. The change in signaling is reported across all trials at each time bin (Figs. 2, 3). To directly compare pulsed trials to continuous wave trials (Fig. 1), PRF pulses were analyzed using the same criterion for success as the continuous wave trials (signal rate increased by either 90% or 0.4 signals per frame compared to its baseline). For consistency, only the 20 s of baseline prior to sonication was included, and only 24 s of sonication was analyzed. 24 s of sonication with the pulsed parameters corresponded to 240 kilocycles which made the comparison between continuous wave (250 kilocycles) and pulsed trials as equal as possible.

### TTX trials

In tetrodotoxin (TTX) trials, the same protocol was used as in pulsed US trials, except that TTX was introduced into the perfusing aCSF at 0 μM, 0.5 μM, or 1.0 μM to serve as a control, or to partially or fully block Na^2+^-channels. Three minutes were allowed for the TTX to diffuse throughout the imaging chamber before running trials. When TTX trials were repeated in a slice, 2 min of rest were given between pulses to allow the baseline to return to normal following sonication.

## References

[1] Fry FJ, Ades HW, Fry WJ (1958). Production of reversible changes in the central nervous system by ultrasound. Science.

[2] Mihran RT, Barnes FS, Wachtel H (1990). Temporally-specific modification of myelinated axon excitability in vitro following a single ultrasound pulse. Ultrasound Med. Biol..

[3] Lee W (2018). Transcranial focused ultrasound stimulation of motor cortical areas in freely-moving awake rats. BMC Neurosci..

[4] Kamimura HAS (2016). Focused ultrasound neuromodulation of cortical and subcortical brain structures using 1.9 MHz. Med. Phys..

[5] Yoo S-S (2011). Focused ultrasound modulates region-specific brain activity. NeuroImage.

[6] Lee W (2016). Image-guided focused ultrasound-mediated regional brain stimulation in sheep. Ultrasound Med. Biol..

[7] Folloni D (2019). Manipulation of subcortical and deep cortical activity in the primate brain using transcranial focused ultrasound stimulation. Neuron.

[8] Yang P-F (2018). Neuromodulation of sensory networks in monkey brain by focused ultrasound with MRI guidance and detection. Sci. Rep..

[9] Deffieux T (2013). Low-intensity focused ultrasound modulates monkey visuomotor behavior. Curr. Biol..

[10] Monti MM, Schnakers C, Korb AS, Bystritsky A, Vespa PM (2016). Non-invasive ultrasonic thalamic stimulation in disorders of consciousness after severe brain injury: A first-in-man report. Brain Stimul..

[11] Hameroff S (2013). Transcranial ultrasound (TUS) effects on mental states: A pilot study. Brain Stimul..

[12] Beisteiner R (2019). Transcranial pulse stimulation with ultrasound in Alzheimer’s disease—A new navigated focal brain therapy. Adv. Sci..

[13] Blackmore J, Shrivastava S, Sallet J, Butler CR, Cleveland RO (2019). Ultrasound neuromodulation: A review of results, mechanisms and safety. Ultrasound Med. Biol..

[14] O’Brien WD (2007). Ultrasound–biophysics mechanisms. Prog. Biophys. Mol. Biol..

[15] Wells J (2007). Biophysical mechanisms of transient optical stimulation of peripheral nerve. Biophys. J..

[16] Shapiro MG, Homma K, Villarreal S, Richter C-P, Bezanilla F (2012). Infrared light excites cells by changing their electrical capacitance. Nat. Commun..

[17] Wattiez N (2017). Transcranial ultrasonic stimulation modulates single-neuron discharge in macaques performing an antisaccade task. Brain Stimulat..

[18] Ye PP, Brown JR, Pauly KB (2016). Frequency dependence of ultrasound neurostimulation in the mouse brain. Ultrasound Med. Biol..

[19] Kubanek J, Shukla P, Das A, Baccus SA, Goodman MB (2018). Ultrasound elicits behavioral responses through mechanical effects on neurons and ion channels in a simple nervous system. J. Neurosci..

[20] Prieto ML, Oralkan Ö, Khuri-Yakub BT, Maduke MC (2013). Dynamic response of model lipid membranes to ultrasonic radiation force. PLoS ONE.

[21] Nikolaev YA, Dosen PJ, Laver DR, van Helden DF, Hamill OP (2015). Single mechanically-gated cation channel currents can trigger action potentials in neocortical and hippocampal pyramidal neurons. Brain Res..

[22] Kubanek J (2016). Ultrasound modulates ion channel currents. Sci. Rep..

[23] Prieto ML, Firouzi K, Khuri-Yakub BT, Maduke M (2018). Activation of Piezo1 but not NaV1.2 channels by ultrasound at 43 MHz. Ultrasound Med. Biol..

[24] Krasovitski B, Frenkel V, Shoham S, Kimmel E (2011). Intramembrane cavitation as a unifying mechanism for ultrasound-induced bioeffects. Proc. Natl. Acad. Sci..

[25] Plaksin M, Kimmel E, Shoham S (2016). Cell-type-selective effects of intramembrane cavitation as a unifying theoretical framework for ultrasonic neuromodulation. eNeuro.

[26] King RL, Brown JR, Newsome WT, Pauly KB (2013). Effective parameters for ultrasound-induced in vivo neurostimulation. Ultrasound Med. Biol..

[27] Tufail Y, Yoshihiro A, Pati S, Li MM, Tyler WJ (2011). Ultrasonic neuromodulation by brain stimulation with transcranial ultrasound. Nat. Protoc..

[28] Kim H, Chiu A, Lee SD, Fischer K, Yoo S-S (2014). Focused ultrasound-mediated non-invasive brain stimulation: Examination of sonication parameters. Brain Stimul..

[29] Legon W (2014). Transcranial focused ultrasound modulates the activity of primary somatosensory cortex in humans. Nat. Neurosci..

[30] Guo H (2018). Ultrasound produces extensive brain activation via a cochlear pathway. Neuron.

[31] Sato T, Shapiro MG, Tsao DY (2018). Ultrasonic neuromodulation causes widespread cortical activation via an indirect auditory mechanism. Neuron.

[32] Yoon K (2019). Effects of sonication parameters on transcranial focused ultrasound brain stimulation in an ovine model. PLoS ONE.

[33] Legon W, Rowlands A, Opitz A, Sato TF, Tyler WJ (2012). Pulsed ultrasound differentially stimulates somatosensory circuits in humans as indicated by EEG and fMRI. PLoS ONE.

[34] Lee W, Kim H, Lee S, Yoo S-S, Chung YA (2014). Creation of various skin sensations using pulsed focused ultrasound: Evidence for functional neuromodulation. Int. J. Imaging Syst. Technol..

[35] Legon W, Ai L, Bansal P, Mueller JK (2018). Neuromodulation with single-element transcranial focused ultrasound in human thalamus. Hum. Brain Mapp..

[36] Morris H, Rivens I, Shaw A, ter Haar G (2008). Investigation of the viscous heating artefact arising from the use of thermocouples in a focused ultrasound field. Phys. Med. Biol..

[37] Nightingale K (2011). Acoustic radiation force impulse (ARFI) imaging: A review. Curr. Med. Imaging Rev..

[38] Menz MD (2019). Radiation force as a physical mechanism for ultrasonic neurostimulation of the ex vivo retina. J. Neurosci..

[39] Tyler WJ (2008). Remote excitation of neuronal circuits using low-intensity, low-frequency ultrasound. PLoS ONE.

[40] Lee J (2019). A MEMS ultrasound stimulation system for modulation of neural circuits with high spatial resolution in vitro. Microsyst. Nanoeng..

[41] Han S, Kim M, Kim H, Shin H, Youn I (2018). Ketamine inhibits ultrasound stimulation-induced neuromodulation by blocking cortical neuron activity. Ultrasound Med. Biol..

[42] Mohammadjavadi M (2019). Elimination of peripheral auditory pathway activation does not affect motor responses from ultrasound neuromodulation. Brain Stimul..

[43] Selzo MR, Gallippi CM (2013). Viscoelastic response (VisR) imaging for assessment of viscoelasticity in voigt materials. IEEE Trans. Ultrason. Ferroelectr. Freq. Control.

[44] Lin Z (2018). On-chip ultrasound modulation of pyramidal neuronal activity in hippocampal slices. Adv. Biosyst..

[45] Morris CE, Juranka PF (2007). Nav channel mechanosensitivity: Activation and inactivation accelerate reversibly with stretch. Biophys. J..

[46] Gaub BM (2020). Neurons differentiate magnitude and location of mechanical stimuli. Proc. Natl. Acad. Sci..

[47] Chen T-W (2013). Ultra-sensitive fluorescent proteins for imaging neuronal activity. Nature.

[48] Schneider CA, Rasband WS, Eliceiri KW (2012). NIH Image to ImageJ: 25 years of image analysis. Nat. Methods.

